# Predictor factors affecting emotional and behavioral problems in school-aged (6–12 years) children during the COVID-19 pandemic: a cross-sectional study

**DOI:** 10.1186/s12888-023-05125-9

**Published:** 2023-08-29

**Authors:** Zohreh Mahmoodi, Mahnoosh Rostami, Giti Bahrami, Fatemeh Rasouli, Nooshin Ghavidel

**Affiliations:** 1 Social Determinants of Health Research Center, Alborz University of Medical Sciences, Karaj, Iran; 2 Health Systems Evaluation and Evidence, Provincial Clinical Excellence, Alberta Health Services, Alberta, Canada; 3 Non-Communicable Diseases Research Center, Alborz University of Medical Sciences, Karaj, Iran

**Keywords:** Behavioral, Corona-related anxiety, COVID-19, Mental health, School-age children

## Abstract

**Objectives:**

The prolonged outbreak of COVID-19 has caused psychological problems in the community, especially in children. Given that limited studies have been conducted on this vulnerable group in Iran, we conducted this study to determine the predictor factors affecting emotional and behavioral problems in school-aged (6–12 years) children during the COVID-19 pandemic.

**Methods:**

We conducted a cross-sectional study on 322 mothers of elementary school-aged children (6–12 years) from April 21 to June 21, 2021, in Karaj, Alborz, Iran. Sociodemographic checklist, socioeconomic status scale (SES), Depression, Anxiety, and Stress Scale (DASS-21), Corona Disease Anxiety Scale (CDAS), and the Rutter Children's Behavior Questionnaire (RCBQ) were used to evaluate the children's behavioral symptoms, the mental health of their mothers, and sociodemographic factors.

**Results:**

In total, 17.7% of children (male = 27.0%, female = 11.7%) had behavioral problems. Results showed income (p-value = 0.007, 95%CI (-3.85- -0.607)), and physical activity of the child (*p*-value = 0.026, 95%CI (-1.03- -0.067)), were indirectly associated with children's behavioral symptoms. Having underlying disease (p-value = 0.00, 95%CI (1.712–5.949)), breastfeeding (p-value = 0.027, 95%CI (0.335–5.61)), corona-related anxiety (*p*-value = 0.00, 95%CI (0.158–0.318)), and DASS score (*p*-value = 0.00, 95%CI (0.149–0.334)) in the mothers, as well as male gender (p-value = 0.003, 95%CI (0.724–3.471)) were directly associated with children's behavioral symptoms. The most factors involved in the behavioral symptom score of children were corona-related anxiety (β = .298), DASS (β = 0.273), underlying disease of mothers (β = 0.163), income (β = -0.154), child's gender (β = 0.142) and physical activity (β = -0.101), and breastfeeding (β = 0.099) respectively.

**Conclusion:**

Study findings showed that some factors affect the emotional and behavioral problems of school-aged (6–12 years) children during the COVID-19 pandemic. These data may help future researchers and policymakers with early intervention.

## Introduction

The SARS-CoV-2 virus pandemic posed a significant challenge to communities worldwide. It had alarming implications for emotional, mental health, and social functioning specially among students [1]. The Iran COVID-19 epidemiology committee, similar to the other countries, declared a state of public health emergency on February 19, 2020, by implementing lockdown measures including, school/childcare closure, restriction on group gathering, temporary suspension of on-essential health and public services as well as border closures and restricted travel [2].

Prolonged outbreaks of COVID-19 caused many challenges in the family, including financial stress, social isolation, emotional experiences of sadness, and loneliness. They also experienced additional parental responsibility due to school/child care closures as they became the lone providers of supervision and education for their children. These challenges and extra pressures affect their mental health, leading to inappropriate behaviors such as nervousness, mood changes, domestic violence, and aggression with their children [3]. In addition, psychological disorders including panic disorder, anxiety, depression, irritability, aggression, and sleep disorders were reported in individuals during the COVID-19 pandemic [4].

While COVID-19 can impact anyone, children may be significantly impacted by the pandemic among other vulnerable groups. This could be due to several factors, including school/childcare closure, playground closure combined with online learning challenges, low COVID-19 media literacy, and coping with new daily routines [1, 4, 5]. Several studies have also reported that pandemics, such as COVID-19, can increase psychological problems in children (e.g., anxiety, depression, Attention Deficit Hyperactivity Disorder (ADHD), post-traumatic symptoms, fear, attachment, and irritability) due to the risk of illness, social isolation, physical distancing, and the increased stress of parents [6–8]. Indeed, emerging evidence highlights that children's behavioral disorders are closely related to parental mental health. Moreover, parents and children with behavioral problems affect each other and other family members [9].

During quarantine, parents are often the closest and best source of help to children. Studies have shown that friendly communication with children is the key to identifying any physical and psychological problems, and also maternal sensitivity was associated with children’s behavioral problems [8, 10]. Although both parents are involved in training the child, the mother has a more significant role in educating the child [11]. Conditions associated with severe stress, emergencies, and natural disasters can increase the risk of psychological complications. Because the mother is one of the essential pillars of life, it can play a critical role in increasing the tolerance of family members against problems. Therefore, mothers' mental health problems have short-term and long-term risks to their children's physical, cognitive, and mental development, affecting parent–child interactions [12].

In addition to family mental health, other factors that can affect children's behavioral problems during an epidemic include household income, reduced outdoor activities, and social interaction [13]. Since lockdown conditions during the COVID-19 pandemic have adverse physical, psychological, and social effects on families, and given that children are very vulnerable to these problems, it is necessary to identify children's behavioral problems, mothers' mental health challenges, and related factors. Then, based on the existing conditions, we can design an intervention to reduce the challenges and issues of the child, parents, and family. Since limited studies have been conducted on this vulnerable group in Iran, this study was conducted to determine the Predictor factors affecting emotional and behavioral problems in school-aged (6–12 years) children during the COVID-19 pandemic.

## Methods

### Design and participants

We conducted a cross-sectional study to explore the factors affecting emotional and behavioral symptoms of elementary school-aged children during the COVID-19 pandemic. We used an online survey to collect data from mothers with a minimum of one child between the ages of 6 and 12 at the study time in Karaj, Alborz, Iran. The inclusion criteria were: Iranian mothers with at least one child between 7 and 12 years old, literate and willing to participate in the study.

Exclusion criteria: the presence of mental illness under psychiatrist treatment in mother or student, the presence of severe chronic diseases such as cancer and multiple sclerosis in mothers, and the presence of behavioral problems in students before the COVID-19 pandemic.

Respondent's decision to complete the survey implied consent to participate in the survey. At the beginning of the study, respondents were informed that the study was confidential and anonymous, and their participation was voluntary. They were also informed that the survey results would be used for presentations or publications. This study was performed by the latest version of the Declaration of Helsinki and with the approval of the research ethics committee of Alborz University of Medical Sciences (IR.ABZUMS.REC.1400.018).

Using multi-stage cluster sampling, we divided Karaj into five geographical districts (North, South, West, East, and Center) and randomly selected 1 or 2 elementary schools from each district. Then we asked the staff of these schools to send the survey link to the parents of the students in all grades. The survey was open from April 21 to June 21, 2021.

### Data collection

Data were completed using a sociodemographic checklist, socioeconomic status scale (SES), Depression, Anxiety and Stress Scale (DASS-21), Corona Disease Anxiety Scale (CDAS), and the Rutter Children's Behavior Questionnaire (RCBQ).

#### Sociodemographic checklist

Sociodemographic checklist contained questions about mothers, including age, occupation, educational level, number of family members, income, number of children, being pregnant, breastfeeding, insurance, history of underlying disease, history of COVID-19 disease, death due to COVID-19 illness in the family or relatives, and the impact of COVID-19 pandemic on mother's job and family income, as well as questions about her elementary school child including sex, student grade, kind of school, living status with parents, BMI (body mass index), and physical activity. For child physical activity, we asked mothers to answer a question, "how many times has your child played sports (Swimming, running, cycling, and aerobics) on the last weekend, in which he/she was very active''. The answers were none, one time, 2- 3 times, 4- 5 times, and six or more times, which were scored from 1 to 5.

#### Socioeconomic status scale (SES)

SES consisted of 6 questions including education of mother and father, income, economic class, and housing status, which are scored based on a Likert scale from 1 to 5, and a total score ranging from 6 to 30. Validity and reliability have been performed in Iran [14].

#### The Rutter Children’s Behavior Questionnaire (RCBQ)

The Rutter Children’s Behavior Questionnaire [15] evaluates the emotional and behavioral symptoms of children aged 6 to 13 years in two forms, one for parents and the other for teachers. We used the parent version, completed by the child's mother and included 31 questions, which were scored based on a Likert scale from 0 to 2 ("does not apply", "applies somewhat", and "certainly applies") and a total score ranging from 0 to 62. It assesses the period of the past twelve months and covers the most frequent emotional and behavioral symptoms. A score of 13 points or more on the total scale was considered to show evidence of a behavioral disorder. This questionnaire has been standardized and validated in Iran (the retest coefficient = 0.90) [16].

#### Corona Disease Anxiety Scale (CDAS)

CDAS was used to measure corona-related anxiety in mothers. This tool has been developed and validated to measure anxiety caused by the coronavirus outbreak in Iran. The final version of this instrument has 18 items, were scored based on a Likert scale from 0 to 3 (Never, sometimes, most of the time, and always) and a total score ranging from 0 to 54. The high scores in this questionnaire indicate a higher level of anxiety in people. The reliability of the questionnaire was reported in 0.919 using the Cronbach's Alpha method in Iran [17].

#### Depression, Anxiety and Stress Scale (DASS-21)

DASS questionnaire was used to assess depression, anxiety, and stress in the mothers [18], consisting of 21 items. Each of the DASS-21 subscales consists of 7 questions, which were scored from 1 to 3 ("does not apply", "applies somewhat", and "certainly applies"). The alpha coefficient for these three factors was reported 0.97, 0.92, and 0.95, respectively [18]. Validity and reliability have also been performed in Iran. Cronbach’s alpha coefficient for the total score of DASS21, Depression, Anxiety and Stress scales were 0.94, 0.85, 0.85 and 0.87, respectively [19].

### Statistical analysis

We used descriptive statistics to analyze survey data using SPSS IBM software, version 20. The mean (SD) of the Children's behavioral symptoms was compared between subgroups of the population using a two-tailed t-test or analysis of variance (ANOVA) and applied the Bonferroni to post hoc multiple comparisons. Correlations between Children's behavioral symptoms with socioeconomic status, DASS, and corona-related anxiety in mothers were measured by Pearson correlation coefficient. Univariable regression analysis was also used to evaluate the association between Children's behavioral symptoms with other variables. A p-value value of less than 0.05 was considered statistically significant.

## Results

In total, 322 mothers completed the survey. The mean age of the respondents was 37.6 (± 5.2) years, and the mean number of family members was 3.8 (± 0.81). The majority of the respondents were unemployed (74.2%), a high school diploma (37.6%), and an income of more than 40,000,000 Rial (53.4%). The COVID-19 pandemic had affected 35.1% of mothers' jobs and 58.7% of family income. The mean (SD) of socioeconomic status was 15.84 ± 4.77 (min 6.0, max 28.0).

The majority of the students whose mothers completed the survey were female (60.9%). The mean (SD) of students' behavioral symptoms was 6.51 ± 7.2, and 17.7% of children (male = 27.0%, female = 11.7%) had behavioral problems. Behavioral problems based on the student's first to sixth grades were 22.1, 15.4, 147, 11.1, 18.3, and 22.6%, respectively. There was no significant difference in behavioral problems between the students' grades (*p*-value = 0.498). The mean (SD) of the BMI of students was 18.58 ± 3.7 (min 8.19, max 35.67). Sociodemographic Characteristics of Subjects have been shown in Table 1.

**Table 1:** Socio-demographic characteristics of subjects

**Variable**			No. (%)
**Mother variables:**	Educational level	< Diploma	52 (16.1%)
		Diploma	121 (37.6%)
		Associate Degree	37 (11.5%)
		Bachelor	77 (23.9%)
		< Bachelor	35 (10.9%)
	Occupational status	Un-employed	239 (74.2%)
		Employed	83 (25.8%)
	Income—Rial	≤ 40,000,000	150 (46.6%)
		> 40,000,000	172 (53.4%)
	History of underlying diseases	Yes	34 (10.6%)
	Pregnant	Yes	10 (3.1%)
	Breastfeeding	Yes	20 (602%)
	Mother's job is affected by COVID-19 pandemic	Yes	113 (35.1%)
	Family income affected by COVID-19 pandemic	Yes	189 (58.7%)
	History of COVID-19 disease	Yes	119 (37.0%)
	Deaths due to COVID-19 Disease in the family or relatives	Yes	148 (46.0%)
**Student variables:**	sex	Male	126 (39.1%)
		Female	196 (60.9%)
	Student grade	First	77 (23.9%)
		Second	26 (8.1%)
		Third	34 (10.6%)
		Fourth	72 (22.4%)
		Fifth	60 (18.6%)
		Sixth	53 (16.5%)
	Single child	Yes	94 (29.2%)
	Kind of school	Public	236 (73.3%)
		Non-profit	86 (26.7%)
	Living with both parents	Yes	299 (92.9%)
	Physical activity last week	None	117 (36.3%)
		1 time	79 (24.5%)
		2 — 3 times	63 (19.6%)
		4 — 5 times	29 (9.0%)
		6 or more times	34 (10.6%)
	Behavioral problems	Yes*	57 (17.7%)
		No	265 (82.3%)

^*^Yes (Possible behavioral problems) is defined as a score of 13 or higher on the total RCBQ scale

Mean (SD) of maternal mental health, including corona-related anxiety, DASS, anxiety, depression, and stress, were 10.48 ± 9.06, 30.64 ± 8.14, 9.29 ± 2.73, 9.79 ± 3.03, and 11.55 ± 3.33 respectively.

We used a two-tailed t-test or ANOVA analysis to evaluate children's behavioral symptoms according to sociodemographic characteristics. The results showed that Children's behavioral symptoms was significantly higher in boys (*p* value = 0.001), studying in non-profit school (*p* value = 0.003), students whose father has died (*p* value = 0.027), and having low physical activity (*p* value = 0.001).

Mothers with educational level > bachelor (*p* value = 0.013), income ≤ 40,000,000 Rials (*p* value = 0.009), underlying chronic disease (*p* value = 0.00), having a single child (*p* value = 0.039), mothers whose job was affected by COVID-19 pandemic (*p* value = 0.001), subjects who had death in the family or relatives due to COVID-19 disease (*p* value = 0.00) had significantly higher behavioral symptoms in their children. The mean (SD) of the children's behavioral symptoms according to sociodemographic characteristics has been shown in Table 2.

**Table 2:** Mean (SD) of the children's behavioral symptoms according to socio-demographic characteristics

**Variable**			**RCBQ score Mean(SD)**	***P*-Value**
**Mother**	Educational level ^a^	< Diploma	5.6 (7.9)	0.013*
		Diploma	6.53 (7.1)	
		Associate Degree	7.57 (7.8)	
		Bachelor	5.03 (5.6)	
		< Bachelor	9.91 (7.8)	
	Occupational status	Un-employed	6.26 (7.1)	0.259
		Employed	7.32 (7.4)	
	Income, Rial	≤ 40,000,000	7.65 (8.01)	0.009**
		> 40,000,000	5.52 (6.3)	
	History of Underlying disease	Yes	13.47 (10.3)	0.00***
		No	5.69 (6.3)	
	Pregnant	Yes	9.1 (10.1)	
		No	6.43 (7.1)	0.25
	Breastfeeding	Yes	10.35 (10.5)	0.10
		No	6.26 (6.8)	
	Mother's job is affected by COVID-19 pandemic	Yes	8.53 (8.5)	0.001**
		No	5.42 (6.1)	
	Family income affected by COVID-19 pandemic	Yes	6.92 (7.66)	0.227
		No	5.93 (6.53)	
	History of COVID-19 disease	Yes	7.04 (7.5)	0.31
		No	6.2 (7.0)	
	Deaths due to COVID-19 Disease in the family or relatives	Yes	8.14 (8.1)	0.00***
		No	5.13 (6.0)	
**Student**	sex	Male	8.16 (7.5)	0.001**
		Female	5.45 (6.8)	
	Student grade	First	6.81 (6.7)	0.90
		Second	5.58 (5.8)	
		Third	6.21 (6.7)	
		Fourth	6.00 (6.0)	
		Fifth	6.65 (9.3)	
		Sixth	7.28 (7.8)	
	Single child	Yes	7.81 (7.2)	0.039*
		No	5.97 (7.1)	
	Kind of school	Public	5.80 (6.9)	0.003**
		Non-profit	8.46 (7.6)	
	The living conditions of the child ^b^	Living with both parents	6.39 (7.0)	0.027*
		Father has died	12.3 (11.4)	
		Divorce of parents	4.84 (5.6)	
	Physical activity last week ^c^	None	8.81 (8.3)	
		1 time	5.51 (6.3)	0.001**
		2 — 3 times	4.73 (6.1)	
		4 — 5 times	5.03 (5.5)	
		6 or more times	5.50 (6.2)	

*RCBQ* The Rutter Children’s Behavior Questionnaire

*a* After the Bonferroni test, children with a mother > bachelor's degree had significantly higher RCBQ score than a mother with a bachelor's degree. *b* After the Bonferroni test, children whose father died had significantly higher RCBQ score than others. *c* After the Bonferroni test, children without physical activity had significantly higher RCBQ score than children with 1, and 2- 3 times physical activity in a week

^*^P-value < 0.05, ** *p*-value < 0.01, *** *p*-value < 0.001, Data were analyzed using a two-tailed t-test or ANOVA

**Fig. 1:**
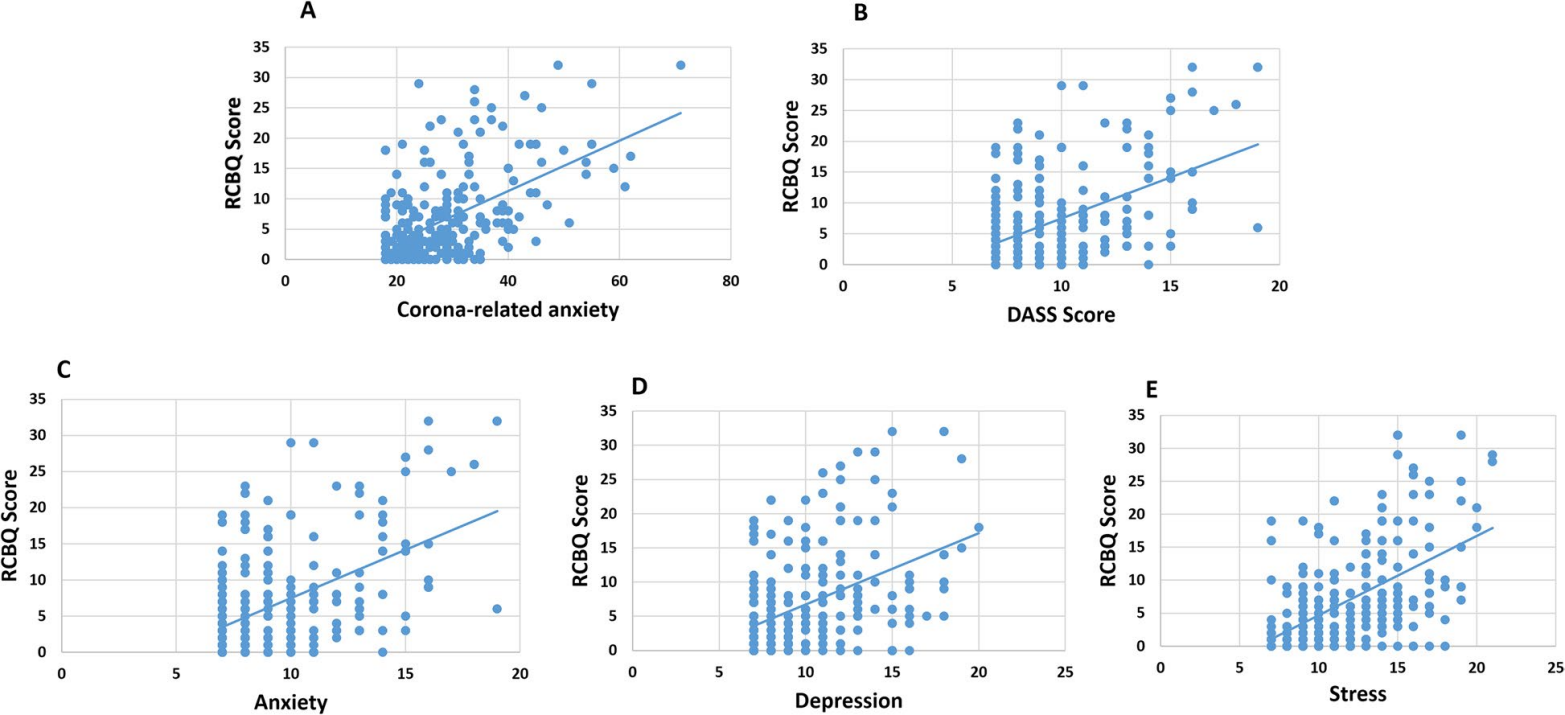
Correlation scatters plots of Rutter Children’s Behavior Questionnaire (RCBQ) and mental health of mothers including corona-related anxiety, DASS, anxiety, depression, and stress. **A** Correlation between RCBQ score and corona-related anxiety (*p*-value = .000, *r* = 0.520); (**B**) Correlation between RCBQ score and DASS (*p*-value = .000, *r* = 0.558); (**C**) Correlation between RCBQ score and anxiety (*p*-value = .000, *r* = 0.505); (**D**) Correlation between RCBQ score and depression (*p*-value = .000, *r* = 0.437), (**E**) Correlation between RCBQ score and stress (*p*-value = .000, *r* = 0.554)

Correlations of children's behavioral symptoms, corona-related anxiety, DASS, anxiety, depression, and stress were assessed using Pearson correlation analyses. Results showed a significant correlation between the children's behavioral symptoms with corona-related anxiety (*p*-value = 0.000, *r *= 0.520), DASS (*p*-value = 0.000, *r* = 0.558), anxiety (*p*-value = 0.000, *r* = 0.505), depression (*p*-value = 0.000, *r* = 0.437), and stress (*p*-value = 0.000, *r* = 0.554) of mothers, but not with socioeconomic status (*p*-value = 0.206) (Fig. 1).

Variables, which had a *p*-value ≤ 0.2 in the univariable model, were entered in the multivariable model, including age, income, number of family members, underlying disease, pregnant, breastfeeding, job and income affected by COVID-19 pandemic, death of a family member or relatives due to COVID-19 disease, socioeconomic status, corona-related anxiety, DASS, as well as sex, school, family condition, and physical activity of the child.

Results showed in the presence of these variables, income (*p*-value = 0.007, 95%CI (-3.85- -0.607)), and physical activity of child (*p*-value = 0.026, 95%CI (-1.03- -0.067)), were indirectly associated with children's behavioral symptoms. Having underlying disease (*p*-value = 0.00, 95%CI (1.712–5.949)), breastfeeding (*p*-value = 0.027, 95%CI (0.335–5.61)), corona-related anxiety (*p*-value = 0.00, 95%CI (0.158–0.318)), and DASS score (*p*-value = 0.00, 95%CI (0.149–0.334)) in the mothers, as well as male gender (*p*-value = 0.003, 95%CI (0.724–3.471)) were directly associated with children's behavioral symptoms (Table 3). The most factors involved in behavioral symptom score of children were corona-related anxiety β = 0.298, DASS β = 0.273, underlying disease β = 0.163, income β = -0.154, male gender β = 0.142, physical activity β = -0.101, and breastfeeding β = 0.099, respectively.

**Table 3:** Association between children's behavioral symptoms with maternal mental health and other variables

Variables		Univariable Model				Multivariable Model^a^			
		Unstandardized Coefficients		*p*-value	Standardized Coefficients	Unstandardized Coefficients		*p*-value	Standardized Coefficients
		B	SE		β	B	SE		β
**Mother**	Age	-0.133	0.078	0.089	-0.095	-.057	.064	.37	-0.041
	Education: > diploma / ≤ diploma	0.545	0.808	0.50	0.038	-	-	-	-
	Job, Employed/ Un-Employed	0.852	0.92	0.35	0.052	-	-	-	-
	Income, Rial, > 40 million/ ≤ 40 million	-2.12	0.80	0.008**	-0.147	-2.23	0.82	0.007**	-0.154
	Number of family members	-1.64	0.49	0.001**	-0.184	-.297	.535	.579	-0.033
	Underlying disease, Yes/ No	7.78	1.238	0.00***	0.331	3.831	1.077	.000***	0.163
	Pregnant, Yes/ No	2.67	2.3	0.2	0.064	-.757	1.847	.682	0.018
	Breastfeeding, Yes/ No	4.09	1.65	0.014*	0.137	2.972	1.340	.027*	0.099
	Infected with COVID-19, Yes/ No	0.84	0.83	0.315	0.056	-	-	-	-
	Mother's job affected by COVID-19 pandemic, Yes/ No	3.11	0.82	0.00***	0.206	.374	.748	.618	0.025
	Family income affected by COVID-19 pandemic, Yes/ No	0.988	0.81	0.2	0.067	-.331	.726	.649	0.023
	Death due to COVID-19 Disease in the family or relatives, Yes/ No	3.015	0.79	0.00***	0.208	1.132	.633	.075	0.078
	Socioeconomic status	-0.107	0.084	0.2	-0.07	.049	.095	.608	0.032
	Corona-related anxiety	0.414	0.038	0.00***	0.52	.238	.041	.000***	0.298
	DASS	0.495	0.04	0.00***	0.558	.242	.047	.000***	0.273
**Student**	Sex, Male/Female	2.705	0.81	0.001**	0.183	2.098	.698	.003**	0.142
	Grade	0.079	0.22	0.72	0.02	-	-	-	-
	Single child, Yes/ No	1.83	0.88	0.039*	0.115	1.186	.914	.195	0.075
	School, non-profit / public	2.66	0.89	0.003**	0.163	.739	.814	.364	0.045
	Family condition: father died/ father alive	5.97	2.3	0.01*	0.144	-.947	1.89	.618	-0.023
	Physical activity	-1.01	0.29	0.001**	-0.185	-.549	.245	.026*	-0.101
	BMI	-0.007	0.10	0.95	-0.003	-	-	-	-

*B* Unstandardized Coefficient, *SE* Standard Error, *β* Standardized Coefficients

^*^P-value < 0.05, ** *p*-value < 0.01, *** *p*-value < 0.001

^a^Adjusted for all variables (with *P* ≤ 0.2 in the univariable model including age, income, number of family members, underlying disease, pregnant, breastfeeding, Mother's job affected by COVID-19 pandemic, Family income affected by COVID-19 pandemic, Death due to COVID-19 Disease in the family or relatives, socioeconomic status, corona-related anxiety, DASS, as well as sex, school, family condition, and physical activity of child

## Discussion

In the current study, we evaluated the factors affecting children's behavioral symptoms aged 6–12 years during the COVID-19 pandemic. Multivariable regression analysis showed that children's family income and physical activity were indirectly associated with children's behavioral symptoms. Having underlying disease, breastfeeding, corona-related anxiety, and DASS score in the mothers and male gender of child were directly associated with children's behavioral symptoms. The most significant factors involved in children's behavioral problems were maternal corona-related anxiety, mental health, and underlying disease, family income, male gender and physical activity of child, and breastfeeding of mothers, respectively.

The result of our study showed that 17% of children had behavioral problems during the corona pandemic, which were at levels previously reported by Khodam and Fatehi [20, 21] for children in the pre-pandemic period in Iran. They examined the prevalence of behavioral disorders in school-age children and used the Rutter parent questionnaire to collect information. Behavioral problems were reported 21% among children in the 4th and 5th grade of primary school in Rafsanjan [20], and 18.4% among school-age children of Gorgan, Iran [21]. Our findings extend to those of Fitzpatrick, who studied children's primary mental health problems and needs during the coronavirus in the United States (US), and found that children's behavioral problems were similar to previous reports [22]. Inconsistent with our study, another study [23] showed significant increases in clinical-level behavioral issues among children and adolescents during the COVID-19 pandemic. This disparity may be due to differences in the study time frame during the corona pandemic, measurement tools, and different conditions of children during the pandemic.

We found that family income was indirectly related to the children's behavioral symptoms, which was in line with other studies. Multivariable regression models in the survey by Vasa [24] indicated that low family income is the risk factor for increased psychiatric problems. As well as other studies showed that behavioral disorders were higher among students in low-income families, and the incidence of behavioral disorders decreased with improving fathers' jobs and family income [25, 26]. Economic stress causes financial conflicts between parents and children, increasing depression in parents, and escalating hostile disputes between husband and wife. These problems can affect children's behavior and developmental processes and cause emotional and behavioral problems in children [4, 27].

Our results showed that children's physical activity was indirectly associated with children's behavioral symptoms. In line with our results, a study in the US showed higher physical activity was associated with fewer externalizing symptoms in younger children during the pandemic. They stated that physical activity effectively promotes health and reduces the impact of the pandemic on children's mental health and well-being [28]. Studies have shown that lockdown restrictions negatively affect the level of physical activity and mental health of children and young adults due to a lack of access to specialized facilities and equipment. Furthermore, children spend less time out of the house, more time sleeping and watching TV during the COVID-19 pandemic [29, 30].

Consistent with other studies, we showed having an underlying disease was directly associated with children's behavioral symptoms. Underlying disease in mothers is a risk factor for children's emotional and behavioral problems in school-age children. The mothers' illness causes distress for children in many areas of their lives [31, 32].

Our study showed breastfeeding experiences directly affected children's behavioral symptoms. A breastfeeding mother may experience physical discomfort and psychological distress [33], such as pain experience, lack of breastfeeding confidence, breastfeeding problems, feeling exhausted, having concerns about breast milk, dealing with relationships, and family problems, which can be frustrating and stressful. Studies have shown that COVID-19 affects breastfeeding plans, leading to specific mental health outcomes. The main reasons reported for its negative impact are the increased childcare responsibilities at home and the lack of family, emotional, and professional support, which have led to an improved experience of anxiety and stress related to breastfeeding [34].

In addition, our results showed that the male gender was directly associated with children's behavioral symptoms, which is in line with existing literature [20, 35]. Studies have shown that males and females have significant differences in the structure and function of the brain, which causes differences in their cognitive and behavioral abilities [35]. However, the study by Khazaee [26] showed no significant differences in children's prevalence of behavioral problems. This disparity may be due to differences in sociocultural circumstances and study population, in which this study was only for first graders.

We found corona-related anxiety and psychosocial problems in mothers were directly associated with behavioral symptoms. In line with our study, the results of a survey by Shirzadi (2020) showed that corona anxiety in mothers had a significant positive relationship with aggression in children [36]. Studies have shown that parents experience cumulative stressors due to COVID-19. The most prevalent stressors among parents included changes to their mood and general stress levels. Conversely, more excellent parental support and perceived control during the pandemic were associated with lower perceived stress and child abuse potential [37, 38]. Similar to our study, a study in Hong Kong on children aged 2–12 years has shown the risk of child psychosocial problems was higher in children who had mothers with mental illness [39]. Furthermore, a study in China has shown that the children of parents with anxiety symptoms were associated with increased risks of emotional symptoms and real difficulty [40]. These results are similar to the study of Hanetz-Gamliel [41] that showed mothers’ anxiety and parenting mediate the associations between COVID-19’s contextual features and children’s behavior problems.

Overall, our study showed that the mental health of mothers had more effect on children's behavioral problems than other factors. These results are consistent with Schiff study [42] which showed that maternal depression and social support play an important role in child's behavioral problems in the context of multiple traumatic events. Mothers are essential pillars of life that can play a critical role in reducing children's challenging behaviors. Parent–child communication helps children and adolescents cope with mental health problems in a public health crisis [43].

This study had several limitations. First, the data was collected online; therefore, some participants didn't access the survey. Second, children's behavioral symptoms were based on mothers' opinions. Third, other factors may be affecting children's behavioral symptoms during the COVID-19 pandemic, which needs to evaluate in other studies.

The strength of this study: Few studies have been conducted in Iran on the behavioral problems of school-aged (6–12 years) children during the COVID-19 pandemic, as well as to our knowledge, no study has been conducted that evaluates several factors including maternal, child and socioeconomic factors together. Therefore, this cross-sectional study identified the predictive factors affecting the emotional and behavioral problems of elementary school children during the pandemic which can help policymakers plan and intervene to improve the mental health of this group of children and their parents.

## Conclusion

Study findings showed that some factors affect emotional and behavioral problems of school-aged (6–12 years) children during the COVID-19 pandemic. The most important factors were corona-related anxiety, mental health and underlying disease of mothers, income, child's gender and physical activity, and breastfeeding of mothers respectively. Given that mothers' mental health was more associated with children's behavioral problems than other factors, these data may help future research and policymakers for early intervention to promote the mothers’ mental health, especially in conditions associated with severe stress such as the Covid-19 pandemic.

## Data Availability

The datasets used during the current study available from the corresponding author on reasonable request.
